# Occupational Therapy and the Use of Technology on Older Adult Fall Prevention: A Scoping Review

**DOI:** 10.3390/ijerph18020702

**Published:** 2021-01-15

**Authors:** María del Carmen Miranda-Duro, Laura Nieto-Riveiro, Patricia Concheiro-Moscoso, Betania Groba, Thais Pousada, Nereida Canosa, Javier Pereira

**Affiliations:** 1 CITIC (Centre for Information and Communications Technology Research), TALIONIS Group, Elviña Campus, University of A Coruna, 15071 A Coruña, Spain; carmen.miranda@udc.es (M.d.C.M.-D.); patricia.concheiro@udc.es (P.C.-M.); b.groba@udc.es (B.G.); thais.pousada.garcia@udc.es (T.P.); nereida.canosa@udc.es (N.C.); javier.pereira@udc.es (J.P.); 2 Faculty of Health Sciences, Oza Campus, University of A Coruna, 15071 A Coruña, Spain

**Keywords:** older adults, falls, occupation, occupational therapy, scoping review, smart home technology, telehealth, exergames, 3D-application tool

## Abstract

*Introduction*: Falls are the second leading cause of accidental or non-intentional deaths worldwide and are the most common problem as people age. The primary purpose of addressing falls is to detect, prevent, treat, and reduce their incidence and consequences. Previous studies identified that multifactorial programs, an interprofessional team, and assistive technology are required to address falls in older adults effectively. Accordingly, the research question is as follows: what are the scope, type of studies, and approaches and strategies to fall risk using technology in the existing occupational therapy literature regarding interventions to address the effects of falls in older adults on daily living? *Methods*: This scoping review was carried out in January 2020 through *Biblioteca Virtual de Salud España*, C.I.N.A.H.L., Cochrane Plus, OTSeeker, PubMed, Scopus, and Web of Science. *Results*: Twelve papers were included. We analyzed the year and journal of publication, authors’ affiliation, and design of the study, and thematic categories. There were three themes: participants’ characteristics, type of intervention, and fall approach and type of technology used. *Discussion and Conclusions*: The literature obtained is scarce. It is considered to still be an emerging theme, especially when considering the use of technology for occupational therapy.

## 1. Introduction

The progressive aging of the population is a well-documented and projected theme of the 21st century, particularly in Europe and Japan [1,2,3]. Certain health conditions associated with the aging process lead to geriatric syndromes, including falls [4]. Current and past scientific evidence has identified falls as the second leading cause of accidental or non-intentional deaths worldwide and the most common and severe problem as people age [5,6,7,8,9,10,11]. Studies have shown that thirty percent of older adults suffer a fall annually, increasing to fifty percent for people aged eighty years and over, and this one of the primary causes of their hospitalization [5,6,7,8,9,10,11]. As people get older, they are more likely to fall. A recent study by Burton and colleagues [12] showed that the prevalence of falls has not changed in the last ten years.

Thus, the primary purpose of addressing falls, identified as a public health challenge, is to detect, prevent, treat, and reduce their incidence and consequences. There is a range of consequences from falls including a reduction in quality of life and increased socioeconomic costs. The consequences on quality of life can include decreased functional mobility, a decreased independence level, social restrictions, depression, loneliness, fear of falling and repeated falls [13,14,15,16,17,18,19].

Several factors can produce falls, and it is relevant to know these to aid in the detection process. According to the World Health Organization (WHO), intrinsic fall factors include factors mainly related to the aging process that affects the person. They also include factors that increased the risk of falls as living alone, taking some medicines (i.e., benzodiazepines) and having some medical conditions (i.e., cardiovascular diseases), impaired mobility and gait, sedentary behavior, fear of falling, nutritional deficiencies, impaired cognition, visual impairments, and foot problems [16]. On the other hand, extrinsic factors occur because older people often have problems with slipping or tripping, lack of good balance, or correction mechanisms to prevent falling. These factors include environmental hazards, unsuitable footwear and clothing, and inappropriate walking or assistive devices [16]. Fear of falling is a syndrome closely related to falls that can produce a fall [16,20]. Moreover, some studies have shown that around one-third of older adults develop a fear of falling after a fall [20,21].

Fall prevention is focused on injuries or complications that occur because of falling [12,22]. Numerous studies [23] have demonstrated that many falls can be prevented through adequate assessment and intervention. Some of the most common and effective interventions include [23] gait stabilizing footwear, vitamin D, dietary supplements, medication adaptation, multiple interventions, multifactorial interventions, assistive device training, cognitive monitoring and intervention, environmental modification, and family and caregiver training [24,25,26,27,28,29].

Different authors have also suggested that multifactorial programs are useful for preventing and reducing falls because of the complexity in the types of falls [30]. According to the World Health Organization (WHO), the risk of falls increases when multiple risk factors are present [16]. These multifactorial programs [30] involve a combination of exercise (focus on strength, balance and mobility), and other options such as individualized and comprehensive fall risk assessment about the person and, their environment and education on fall prevention. The GeriaTIC project is an example of using multifactorial interventions for fall prevention [31]. A study by Close and colleagues highlighted that an interprofessional approach to this high-risk population can significantly decrease the risk of further falls and limit functional impairment [32].

The effectiveness of these multifactorial programs is also based on the need to have an interprofessional team for fall prevention and treatment and geriatric approaches [33]. This includes a physician, a neurologist, a nurse, a psychiatrist, a physical therapist, and an occupational therapist. The latter is part of the non-pharmacological intervention [34,35]. Elliot and colleagues [36], in a systematic review, classified occupational therapy interventions on older adults’ falls into three types of intervention that are shown in Table 1.

In addition to multifactorial programs and the interprofessional team, more emphasis is being placed on technology use. Technology has been integrated into some of the interventions for, or approaches to, fall reduction [18]. Assistive technology has been used to enable and promote inclusion and participation, maintain or improve functioning and independence, and promote well-being and active living [41,42,43].

According to the WHO [41], around the world, there are one billion people who require assistive products today. More than two billion people worldwide are expected to need at least one assistive product by 2030 [41]. Some examples of using assistive technology in older adults’ falls interventions may include, video monitoring, health monitoring, electronic sensors, and equipment such as fall detectors, door monitors, bed alerts, pressure mats, and smoke and heat alarms, according to Miskelly and colleagues [42].

Also, remembering that exercise is the most common approach to falls, previous European projects such as the iStoppFalls [44], Farseeing [45] and Prevent IT [46] project were focused on using technology to improve older adults’ physical functioning.

Accordingly, this study’s primary goal is to explore the scope of occupational therapy literature regarding interventions to address the effects of falls in older adults on daily living with technology. Specifically, we intend to:

- Describe the types of studies on this topic and where they are usually published;
- Describe proposed occupational therapy approaches and strategies to fall risk using technology.

## 2. Materials and Methods

**Figure 1:**
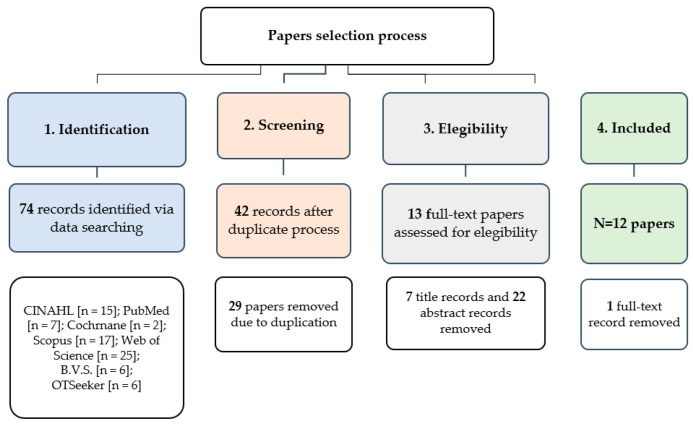
Flow diagram of the Scoping Review process.

The authors conducted a scoping review in January 2020. The research questions that we aimed to answer were the following: what are the scope, type of studies, and approaches and strategies to fall risk using technology in the existing occupational therapy literature regarding interventions to address the effects of falls in older adults on daily living? Two approaches guided the present scoping review. On the one hand, the Arksey and O’Malley [47] five-stage framework was used, which includes stage 1 establishment of the research question; stage 2 identification of pertinent studies and choice of studies, stage 3 study selection as explained in Figure 1; and, as shown in the Results section, the stage 4 charting the data and stage 5 mapping the data and collating, summarizing, and reporting the findings. On the other hand, this scoping review also follows the Preferred Reporting Items for Systematic reviews and Meta-Analyses extension for Scoping Reviews (PRISMA-ScR) (see Table S1 [48]. In accordance with the aim of a scoping review, a quality appraisal is not required, as opposed to Systematic reviews and Meta-Analyses [49].

### 2.1. Sources and Search Strategy

The search was focused on seven electronic databases (see supplementary Table S2 in the supplementary materials), specifically on health sciences and occupational therapy: *Biblioteca Virtual de Salud España* [50], C.I.N.A.H.L. [51], Cochrane Plus [52], OTSeeker [53], PubMed^®^ [54], Scopus [55], and Web of Science [56].

The criteria used were flexible due to this being a current topic. The eligibility criteria were papers written in English, Portuguese, and/or Spanish; there was no limit on the year of publication; studies could only involve humans; and all types of documents (i.e., original articles, reviews, conference papers) were considered. Individual search strategies (see supplementary Table S2 in the supplementary materials) were used for each database using a combination of the operators “AND” and “OR,” jointly with the criteria defined and the following descriptors:

- occupational therapy, ergotherapy;
- falls, accidental falls;
- aged, geriatrics, gerontology, older adult, older person, elder person, older people, elderly, elderly people, veteran, retired, senior;
- technology, technologies, wearable electronic devices, wearables, computers, digital games.

### 2.2. Study Selection and Data Extraction

After searching, in accordance with the PRISMA-ScR guidelines [48], the first step was to identify all of the records, which were imported into the bibliographic manager Mendeley [57]. The second step was screening, whereby duplications were removed through Mendeley [57]. In the eligibility step, the results were assessed by title, abstract, or full text, following the eligibility criteria defined and in accordance with the topic of interest—occupational therapy interventions based on technology to address falls in older adults (see supplementary Table S3 on supplementary materials). Thus, the studies had to involve falls, the older adult population, an occupational therapy perspective, and the use of technology. In total, 12 papers met the defined criteria. See the details of these processes in the Figure 1 in the Section 3.

### 2.3. Data Analysis

Data were compiled in Microsoft Excel^®^ (Redmond, DC, USA) for validation, coding, and analysis. Bibliometric and thematic variables were used to analyze the characteristics of the studies. Frequencies and/or percentages were used to show the following bibliometric characteristics: year of publication, authors’ affiliation, journal of publication, and type and design of the study. A thematic analysis was also conducted as a “*method for identifying, analyzing, and reporting patterns (themes) within data*” ([58], p. 79). According to Braun and Clarke “a theme captures something important about the data in relation to the research question, *and represents some level of patterned response or meaning within the data set*” ([58], p. 80). For this reason, the authors identified the following three themes: a description of the type of participants in study, types of intervention and approach to falls, and type of technology used.

## 3. Results

A total of 12 papers met the eligibility criteria and were included in the present Scoping Review. Figure 1 gives an explanation of the selection process for choosing these papers. Table 2 and Table 3 summarizes the papers.

### 3.1. Bibliometric Characteristics

In total, we screened 74 documents, with 12 studies meeting the eligibility criteria (see Figure 1). The selected literature was published between 2012 and 2020. The first study to report the use of technology in an occupational therapy falls approach was published in 2012 [58].

The studies were conducted in the USA (*n* = 4) [58,59,60,61]; Europe (*n* = 6) [62,63,64,65,66,67], specifically in Scotland (*n* = 1) [62], Sweden (*n* = 1) [63], the UK (*n* = 3) [64,66,67], and Belgium (*n* = 1) [65]; and other countries such as Tunisia (*n* = 1) [68] and Australia (*n* = 1) [69].

The journals in which the articles were published were mainly those focusing on occupational therapy (*n* = 6) [58,59,60,61,62], particularly for the first publications of studies on the topic, and informatics and technological journals (*n* = 6) [64,65,66,67,68,69], which contained more of the recent studies.

The types of study were reviews—a systematic review (*n* = 1) [58] and a critical review (*n* = 1) [59] and original articles (*n* = 10) [59,60,61,67,68]. Within the original articles, there were different research designs. Quantitative approaches were used, including a descriptive study (*n* = 1) [66], a case study (*n* = 2) [60,61], and an experimental study (*n* = 1) [68]. The qualitative approaches used were qualitative research (*n* = 2) [63,69] and a mixed-methods study (*n* = 2) [65,66].

**Table 2:** Summary of data extracted from the 12 selected studies.

Author(s), Year, [Reference]	Authors’ Affiliation	Journal of Publication	Type of Study and Purpose	Sample Characteristics (Size, Age, % of Female, Setting, and Others)	Technology Used	Main Findings
Chase, C.A.; Mann, K.; Wasek, S. and Arberman, M. 2012 [58]	Western Michigan University; Rehabilitation Hospital of Indiana; Ingham County Medical Center and Rehabilitation; University of Buffalo. U.S.A.	*Am. J. Occup. Ther.*	This systematic review aims to synthesize existing literature about the effect of home modification as both a separate intervention and a component of several fall prevention programs.	*n* = 33 studies, including a total of 31 randomized controlled trials	Commercially available smart home technology: operate lights, appliances, door, and windows for frail older adults living alone.	The results contribute to evidence-based practice for occupational therapy practitioners working with older adults in community-based settings and reinforce the importance of the role of occupational therapy in the home and community.
				Age = is focused on older adults (not specify ages)		
				Setting = community-dwelling older adults		
				Female = Number of females not specified		
Stewart, L. and McKinstry, B. 2012 [62]	Astley Ainslie Hospital; Edinburgh University, Edinburgh	*Br. J. Occup. Ther.*	This critical review aimed to evaluate the association between older people’s fear of falling and the use of telecare and whether telecare could reduce this fear.	*n* = 10 studies, including randomized controlled trials, a cohort study, two qualitative studies, a case study, and surveys	Telecare: this is understood as the remote or enhanced delivery of health and social services to people in their own homes using telecommunications and computerized systems.	Telecare’s contribution to supporting an aging population at home for longer is becoming increasingly recognized by health and social care services worldwide. However, this critical review identified that few studies are investigating older people’s views and the use of telecare in the domain of occupational therapy.
				Age = older adults over 60 years old		
				Setting = older adults from public-assisted housing, day centers, and community living		
				Female = Number of females not specified		
Horowitz, B.; Nochajski, S.M. and Schweitzer, H.A. 2013 [59]	York College- CUNY; University of Buffalo; State University of New York. U.S.A.	*Occup. Ther. Health Care*	This case study focused on the development and pilot-testing of the Home Safety Self-Assessment Tool (H.S.S.A.T.), a new home assessment tool designed for use by older adults to promote home safety and aging.	*n* = 28 older adults	Videos: developed with instructions to install home modifications to prevent falls. Several tools were included to analyze the risks associated with older adults’ homes in the project.	The results suggest the tool may assist older adults in identifying environmental factors related to falls and facilitate their ability to age in place.
				Age = between 69 to 87 years old		
				Female = 68%		
				Setting = community dwelling		
Charness, *n*. 2014 [60]	Florida State University. U.S.A.	*Occup. Ther. Health Care*	This case study was used to illustrate telehealth as one important tool to improve the efficiency of healthcare delivery.	*n* = 9 older adults	Telehealth system: consisting of a watch for a factor sensor system that monitors temperature with an analog display, an emergency button, and an accelerometer to provide information about activity and to monitor falls.	Telehealth systems can provide potentially important support for persons to live independently longer through automated monitoring. The purpose of this study was to find a cost-effective telehealth technology.
				Age = older than 75 years		
				Female = Number of females not specified		
				Setting = community-dwelling		
Bianco, M.L.; Pedell, S. and Renda, G. 2016 [69]	Swinburne University. Australia	*A.C.M.*	This qualitative research study assessed the perceptions of ten older adults on an augmented reality tool.	*n* = 10 older adults	An augmented reality application prototype on an iPad. The application is a 3D model library bank of typical and novel home modifications. Professionals can access this modification bank to superimpose a proposed recommendation into their client’s home environment for evaluation and discussion.	The findings indicate that many older adults welcome augmented reality as a design and communication medium. It can be used as a bridging mechanism to increase the person-centeredness of fall prevention services.
				Age = between 69 and 92 years old with a mean age of 79.1 years		
				Female = 60%		
				Setting = not specified		
Glannfjord, F.; Hemmingsson, H. and Larsson, A. 2016 [63]	Linkoping University. Sweden	*Scand. J. Occup. Ther.*	This qualitative (phenomenology) study examined how older adults perceive the Wii, namely the Wii sports bowling game, in an activity group.	*n* = between 10 and 12	The Nintendo Wii sports bowling game, with the Wii controller, was compared with real-life bowling.	The Wii was found to be an enjoyable and social activity. The interviewers looked forward to participating in the activity each week. Participants felt like they were really bowling in this virtual activity, and positive differences between regular bowling and virtual bowling were identified; virtual options were identified as being easier.
				Age = mean age of 78, between 64 and 98 years old		
				Female = Number of females not specified		
				Setting = Activity center for elderly people		
Hamm, J.; Money, G.A. 2017 [64]	Brunel University, London South Bank University. U.K.	*Health Informatics J.*	This mixed-study explored occupational therapists’ perceptions of an early-stage, three-dimensional measurement aid prototype, which provides enhanced assistive equipment provision process guidance to clinicians.	*n* = 10 occupational therapists	A 3D measurement aid prototype (3D-MAP) application, using 3D visualization technology was deployed on a tablet, mobile phone, or laptop. This was based on the five most commonly measured items with the Assistive Equipment Provision Process tool (bed, bath, toilet, chair, and stairs).	The results show that occupational therapists considered that the 3D-MAP application could effectively augment existing 2D diagrams and deliver numerous benefits.
				Female = 100%		
				A ten pounds voucher was offered 2 to 31 years of experience.		
				The occupational therapists’ experience was in adults, social services, surgical rehabilitation, neurology, re-ablemen, and social services.		
Lemmens, R.; Gielen, C. and Spooren, A. 2017 [65]	P.X.L. University College. Belgium	*Stud. Health. Technol. Inform.*	This was a qualitative study about developing a screening tool to enable occupational therapists to assess people’s home environments to facilitate independent living.	1st phase:	The Obstacle tool is a digitalized version using the mind maps and the paper version results for tablets. It has a version that can be used by health professionals or informal caregivers too. The digital version includes (1) the possibility of structuring the screening by adding the rooms in the order of preference for occupational therapists, (2) registration of problems/scores, (3) the addition of photos to the screening, (4) a better overview than in the paper version, (5) the option to store and save data and make a back-up, and (6) connection with the application H-OPP (a digital coach for occupational therapists).	The Obstacle tool was developed and judged to be very useful by occupational therapists. It was highlighted that the Obstacle is adapted for use for persons with dementia and a mini-obstacle tool is under construction and will be digitalized to be available for clients and their informal caregivers. The next step is to make the tool accessible to everybody.
				*n* = 16 older adults in their homes		
				Aged over 65 years old		
				2nd phase:		
				*n* = 31 older adults		
				Aged over 65 years old		
				3rd phase:		
				*n* = 5 older adults, 5 informal caregivers, 5 professional caregivers		
				Setting = community-dwelling		
				The number of females was not specified		
Arthanat, S.; Wilcox, J. and Macuch, M. 2019 [61]	The University of Hampshire. U.S.A.	*O.T.J.R.*	This descriptive study aimed to determine the extent to which smart home technology has been adopted by older adults, what types of smart home devices are being used, the health factors related to the adoption of this technology, and the factors that contribute to smart homeownership and readiness.	*n* = 445 older adults	Smart Home Technology: sensor networks to monitor and gather information about the state of the home and its residents, mechanisms that allow communication between devices to enable automation and remote access, and user interfaces such as home displays, personal computers, tablets, and smartphones to enable consumers to set preferences/goals and receive information and feedback.	The present study concluded that adoption and interest in smart home technology are relatively low among older adults. The levels of ownership and readiness vary vastly by technology, demographic segments, functional status, and home safety. These aspects could be taken into account by occupational therapists.
				Age = a mean age of 70.7, between 60 and 95 years old		
				Setting = community dwelling		
				Female = 68%		
Hamm, J.; Money, A.G. and Atwal, A. 2019 [66]	Brunel University, London South Bank University. U.K.	*J. Biomed. Inform.*	This mixed-method study aimed to present a 3D mobile application to enable older adults to carry out self-assessment measurement tasks in accordance to two different treatment conditions, using a 3D guidetomeasure tool or a 2D paper-based guide.	*n* = 37 participants	The application 3D guidetomeasure-3D was developed by the Unity3D game engine, which supports multi-platform deployment, including Android, IOS, desktops, and Web. The unity3D engine includes an avatar model, 3D furniture models, and arrow prompts of the application.	An empirical mixed-methods assessment of the performance of the guidetomeasure-3D application revealed that, in terms of accuracy, consistency, task completion time, and usability, significant performance gains were achieved over the art’s current state paper-based 2D measurement guide equivalent.
				Age = mean age of 68.5, between 55 and 86 years old (20 retired, 11 employed, three not specified)		
				Female = Not specified		
				Setting = Not specified		
Money, A.G.; Atwal, A.; Boyce, E.; Gaber, S.; Windeatt, S. and Alexandrou, K. 2019 [67]	Brunel University U.K.	*B.M.C. Med. Inform. Decis. Mak.*	This mixed-method study used Falls Sensei 3D to evaluate the overall game usability from an older adult perspective and to explore older adults’ perceptions of using Falls Sensei, the factors affecting the adoption of this application, and the extent to which the modification of fall-prevention-related behavior can occur as a consequence of playing the Falls Sensei game.	*n* = 15 participants	Falls Sensei 3D game is a first-person 3D exploration game with four levels that correspond to four key living areas within the home: the kitchen, bathroom, bedroom, lounge, and stairs. The application was developed with Unity3D to generate a GameObject, which contains 3D Models and associated scenes presented at each game level.	This study offers a promising exploration into using challenging games to address extrinsic factors in fall risk reduction. Data analysis triangulation suggests that the game raised awareness of home hazard detection, but further research is needed to draw comparisons with established interventions.
				Age = between 50 and 80 years old		
				Setting = adults attending a 50s gym group on a university campus		
				Female = 60%		
Haj, A. B. and Khalfallah, A. 2020 [68]	The University of Sfax. Tunisia	*Smart Innovation, Systems, and Technologies. Book Series*	This experimental study aimed to propose an exercise to improve patients’ posture with the use of Kinect.	One older adult	Kinect is designed to control video games while allowing human–machine interaction without markers or a joystick. The body interacts with the machine. Kinect allows the acquisition of RGB video, a depth map, and sound through the libraries supplied with the software kit. Kinect includes two cameras—RGB and depth, a 3D camera that enables 3D motion capture, and a microphone.	This study has limitations because the Kinect sensor has limitations in precision compared with other more expensive motion 3D sensors. The authors recommend that this exercise is used by older people who do not suffer from dizziness, neurological disorders, or severe pathological diseases.
				No details were specified		

### 3.2. Thematic Categories

#### 3.2.1. A Description of the Type of Participants in Study

This theme was about the types of participants included in the studies (i.e., older adult(s) or occupational therapist(s)), the age of the participants, the percentage of females in the sample, the environment in which the study was conducted (i.e., community-dwelling or an institution), and other interesting details mentioned.

The reviews included in this study did not provide some of the participants’ characteristics (see Table 3 details of the participants). In a review by Chase and colleagues [58], a total of thirty-three studies were included, all randomized controlled trials, while in the review by Stewart and colleagues study [62], ten studies were included and these were different types of studies.

The original articles [59,60,61,63,64,65,67,68,69] included 614 older adults, 15 occupational therapists, and 5 informal caregivers. The samples size of the studies included 1 (*n* = 1) [68] to 445 (*n* = 1) older adults [61], 1 (*n* = 7) [59,60,61,63,66,67,69] to 10 (*n* = 1) types of occupational therapy [64], and 1 (*n* = 9) to 5 (*n* = 1) informal caregivers [65]. There was a large age range age of older adults from 50 years old (*n* = 1) [67] to a maximum of 98 years old (*n* = 1) [63]. These people were recruited from the community (*n* = 8) [59,60,61,65], an activity center for older adults (*n* = 1) [63], or people attending a gym group on a university campus (*n* = 1) [67]. Regarding the percentage of females, all studies included more females than males, with a percentage of females of between 60 (*n* = 2) [64,69] and 68% (*n* = 2) [59,61]. One hundred percent of the occupational therapists were female [64].

All participants volunteered to participate in the different studies, but in the study by Hamm and colleagues, the occupational therapists received a ten pound voucher [64].

#### 3.2.2. Type of Intervention and Approach to Falls

This theme included the type of intervention if the study was about a specific risk factor related to falls (extrinsic, alien to the individual, or intrinsic, related to the person and the aging process) and the type of approach to falls (detection, prevention, or treatment).

Four types of intervention were identified: “home modifications” (*n* = 5) [59,64,65,66,69], “assistive technology” (*n* = 4) [58,60,61,62], “exercise” (*n* = 2) [63,68], and “educational” (*n* = 2) [59,67]. We considered single-component interventions and focused on fall detection, prevention, and treatment. Interventions that focused on home modification addressed extrinsic factors such as bathroom modifications (i.e., bath, toilet, shower), appropriate chair height, and indications to include space to move, among others. Assistive technology interventions addressed extrinsic factors that can affect the safety of the person in the home. This included the use of telecare, emergency alarms, and fall detectors (i.e., pendant alarms). Exercise was used as a strategy to address intrinsic factors related to physical condition and the use of educational interventions in these studies was based on extrinsic factors, which consisted of the types of modification the person has to do to be safe at home.

#### 3.2.3. Type of Technology Used

This theme was about the types of technology addressed in the studies. The technologies used in the studies were classified as software developments, telehealth, multimedia materials, and commercial and technological devices. Software developments (*n* = 5) included augmented reality applications [69], 3D measurement aid prototype applications [64,66], a digital version of the Obstacle Tool [61], and a Falls Sensei 3D game [67]. The use of telehealth was integrated into telecare to reduce the fear of falling [62], and the system was integrated into a watch to provide a factor sensor system to monitor temperature with an analog display, an emergency button, and an accelerometer [60]. The multimedia materials included different videos to identify environmental fall factors [59]. The commercial and technological devices included smart home technology to operate lights, appliances, doors, and windows [58], and these included the use of Kinect [68] and Nintendo Wii [63].

Table 3 shows the relationships between Section 3.2.2 (Type of intervention and approach to falls) and Section 3.2.3 (Type of technology used, and the compatibility of the technology used with the technological devices: Tablet, iPad, computer, laptop, Xbox, Nintendo Wii, and mobile phone).

**Table 3:** Relationships between Section 3.2.2. and Section 3.2.3.

Types of Technology	Technology Used	Compatibility	Intervention	Falls Risk
Software developments	Augmented reality application	iPad	Home modifications	Extrinsic factors
	3D measurement aid prototype application	Tablet, mobile phone, or laptop		
	Digital version of Obstacle Tool	Tablet		
	Falls Sensei 3D game	Computer		
Telehealth	Telehealth system	Not applied	Assistive technology	Extrinsic factors
	Telecare			
	Smart home technology			
Multimedia materials	Videos	Computer, laptop, mobile phone or Tablet	Educational	Extrinsic factors
Commercial and technological devices	Kinect with Xbox and Nintendo Wii	Kinect with Xbox and Nintendo Wii	Exercise	Intrinsic factors

## 4. Discussion

This study presents the first scoping review of occupational therapy interventions to address older adults’ falls using technology. The objectives defined focused on exploring the literature about the topic to determine the type of studies conducted, where these studies have usually been published, and the approaches and strategies used to reduce fall risk by occupational therapists using technology.

The results show that this is an emerging area, which began to be researched in the year 2012 [58]. In a review by Chase and colleagues on home modification, only telecare was mentioned as a possible strategy by occupational therapists, but occupational therapists did not specifically develop that study. However, previous studies focused on the use of technology. For example, the iStoppFalls project focused on the use of exergames to reduce falls in older adults; this project was conducted from 2011 to 2014 to motivate and enhance the use of physical activity by community-dwelling adults aged more than sixty-five years by engaging with three purpose-built exergames to reduce falls [43,70].

The present review shows a trend toward carrying out studies with a qualitative approach [63,69] and mixed-methods studies [65,66], reinforcing the idea that is important to understand the perceptions and opinions of the older adults or occupational therapists and other health professionals under study to find out about their experiences with using the technology. This type of research helps us to understand the acceptance or not of technology and to determine how to improve or adapt it to make it useful in older people’s day-to-day lives [71].

Regarding the place of publication, similar numbers of studies have been carried out in the USA [58,59,60,61] and Europe [62,63,64,65,66,67], even though progressive aging of the population is more apparent in Europe, and considering that previous European projects such as Prevent IT, Farseeing, and iStoppFalls, which are an essential background to fall prevention and the use of technology, were developed in Europe [43,44,45].

Compared with other reviews about falls and occupational therapy [41,69], the present scoping review results are of a lower research quality, because it was not possible to find any controlled trials to demonstrate the effectiveness of the interventions developed alongside technology. The types of studies included descriptive studies [61], case studies [59,60], experimental studies [68], and qualitative studies [63,69], which are not considered to give a high level of evidence. Instead, some controlled trials were carried out in the field of falls and occupational therapy, for example, the one by Monaco and colleagues [39].

As for the participant sample sizes and characteristics, the samples used were relatively small, except for one case [61]. This is linked to the level of evidence mentioned above and the types of study used. The types of participant included older adults, occupational therapists, and/or informal caregivers.

Only one of the types of software developed, obstacle tool digitalization [65], was tested in older adults, occupational therapists, and informal caregivers. The aim was to make it accessible for everybody, which is an essential factor to keep in mind in software development, according to the accessible software development model [72], the philosophy of design for all [73], and the inclusive perspective of the occupational therapy [74], as this helps to break the digital divide, particularly among older adults [74].

The studies included an extensive range of ages from 50 [67] to 98 years old [63], although older persons are classified as those aged 65 or more years. This reflects the perspective of preventing falls in people nearing retirement and the importance of active, healthy aging throughout life [75,76]. The life expectancy in Europe and USA, the main places of publication, is approximately 82 years old [74]. Life expectancy at age sixty is higher in women than men [77], and as can be seen in the results, females made up the highest percentage of participants in the studies, with 60–68% of participants being older women [67,69].

Regarding the type of intervention used to address falls in older adults through occupational therapy, only single-component interventions were used [36], even though different authors have suggested that multifactorial programs help to prevent and reduce falls because of their complexity [30]. None of the studies included an interprofessional team, despite its importance. The effectiveness of the multifactorial programs is also due to the use of an interprofessional team for fall prevention and treatment [33].

In terms of multifactorial programs [30], these involved exercise, as was done in two studies included in this review [63,68]; individualized and comprehensive fall risk assessment about the environment of an older adult, as done in a few studies mentioned in this review [59,64,65,66,69]; and education on fall prevention, as done in two studies [60,68]. Furthermore, any intervention includes occupational therapy home visits [78], which can be an essential aspect to include, especially in the cases of home modification [59,64,65,66,69] and assistive technology [58,60,61,62]. The studies that integrated aging in place focused on assistive technology and home modifications because these factors are widely acknowledged as being the primary and preferred interventions during ageing [79,80]. Understanding how older people’s needs contribute to improving their quality of life, which is affected after a fall, is necessary [13,14,15,16,18,19].

A previous systematic review explored the cost-effectiveness of several occupational therapy interventions for older people, concluding that they are useful and cost-effective compared with standard care or other therapies [81]. In this way, socioeconomic impact is one of the consequences of falls [28]. However, the results were not focused on aspects related to socioeconomic impact.

Our results reinforce the idea that home modifications, assistive technology, and educational interventions can address extrinsic factors, particularly environmental factors, and exercise can address intrinsic factors. This is in accordance with previous studies about the use of occupational therapy interventions to address fall risk [24,82,83,84,85].

Although, as mentioned above, some examples of assistive technology used to prevent falls in older adults include video-monitoring, health monitoring, electronic sensors, and fall detectors [42] these were not included in the studies mentioned in this review. Moreover, regarding fall interventions globally, there is more focus on exercise options and an extensive range of technologies from virtual reality [86] to wearables [87] that were not included in these studies. Primarily, virtual reality interventions are used in occupational therapy, for example, in children [86].

As a result of this review, it is suggested that researchers in this field perform more studies that include the latest technology in the field of falls, so that more studies with a higher level of evidence exist. Interprofessional and multifactorial interventions should be integrated.

### Limitations

The present scoping review has few limitations since all those publications related to the topic have been included; regarding the language were included articles in English, Spanish, and Portuguese; and regarding the type of study, any of them were included. The first limitation maybe not including other languages or other databases in the search process. However, we included databases of occupational therapy and socio-health care. Regarding the searches and the inclusion and exclusion of studies, it was carried out by one of the authors, which may be a limitation. In spite that the researcher used a structured process, some data may have been omitted or excluded. As future research, it would be important to integrate more researchers into this process.

## 5. Conclusions

Although falls have been identified as a public health challenge and the importance of technology in our lives is well known, the literature available on the prevention of falls in older adults using technology is scarce. It is considered to be an emerging area, especially when considering the use of technology in occupational therapy.

The studies in this area have mainly been conducted in the USA and Europe and have been published in occupational therapy and informatics journals. The target population is those over 50 years of age. The risk factors that have most frequently been evaluated and considered are extrinsic factors, particularly environmental factors. Interventions on occupational therapy using technology to address falls in older adults have been single component methods, including home modifications, assistive technology, educational intervention, and exercise. The technology used in the studies can be classified as software developments, telehealth, multimedia material, and commercial technological devices. Lastly, the authors conclude that the prevention of falls in older adults is an essential part of interventions against the risk of falls, and occupational therapy and the use of technology may contribute greatly to interprofessional fall prevention programs.

## Figures and Tables

**Table 1:** Summary of occupational therapy interventions for falls in older adults.

	Summary of Occupational Therapy Interventions for Falls in Older Adults
**Single component** (includes only one of the following components)	Exercise
	Home safety assessment
	Education about falls’ prevention
	Example: Lifestyle Integrated Functional Exercise study [37]
**Multicomponent intervention** (includes exercise and one of the following options)	1st option: educational components as:
	Feet or footwear risk
	Energy conservation strategies
	Safe assistive device use, home modification
	Fall recovery
	Medication routines
	Nutrition and hydration
	Relaxation stress management
	2nd option: home modification with other fall prevention intervention
	Example: Minimally Supervised Multimodal Exercise to Reduce Falls Risk in Economically and Educationally Disadvantaged Older Adults [38]
**Multifactorial intervention** (include the complex assessment of different components)	Fall risk
	Environment, education, and group activities
	Activities of daily living
	Assistive devices
	Self-efficacy or fear of falling
	Example: A single home visit by an occupational therapist reduces the risk of falling after hip fracture in elderly women: A quasi-randomized controlled trial [39]
**Population-based fall prevention** (includes strategies implemented across whole communities, two different types)	Existing effective population-based fall prevention programs
	Other population-based multicomponent interventions
	Example: Stepping On -Translating a Fall Prevention Intervention Into Practice: A Randomized Community Trial [40]

## Supplementary Materials

The following are available online at https://www.mdpi.com/1660-4601/18/2/702/s1, Table S1: Preferred Reporting Items for Systematic reviews and Meta-Analyses extension for Scoping Reviews (PRISMA-ScR) Checklist. Table S2: Search strategies from each database, Table S3: Removed from eligibility criteria.
