# Supplementary material for: Occupational Therapy and the Use of Technology on Older Adult Fall Prevention: A Scoping Review
Source: Int J Environ Res Public Health. 2021 Jan 15;18(2):702. doi: 10.3390/ijerph18020702 (PMC7830762; doi:10.3390/ijerph18020702)
Supplement: Supplementary file 1 [file ijerph-18-00702-s001.zip › ijerph-1017994-ES-SI-supplementary/Supplementary tables S2-3.docx]

**Supplementary materials**

**Table S2.** Search strategies from each database.

| **Database** | **Search Term Used** | **Results** | **Date** |
| --- | --- | --- | --- |
| CINAHL | ( ( “Occupational Therapy” OR ergotherapy) AND ( “Accidental Falls” OR “Falls” ) AND ( “Aged” OR “Geriatrics” OR “Older Adult” OR “Older Person” OR “Elderly” OR “Elderly People” OR “Veteran” OR “Retired” OR “Senior” OR “Older People” OR “Elder Person” OR “Gerontology” ) AND ( “Technolog*” OR “Computers” OR “Wearables” OR “Digital games” ) ) Language = English | 15 | 08/01/2020 |
| PubMed | (((“Occupational Therapy”[Mesh] OR “Occupational therapy”[Title/Abstract] OR ergotherapy [Title/Abstract])) AND (“Accidental Falls”[Mesh] OR “Accidental Falls”[Title/Abstract] OR “Falls”[Title/Abstract])) AND (“Aged”[Mesh] OR “Aged”[Title/Abstract] OR “Geriatrics”[Mesh] OR “Geriatrics”[Title/Abstract] OR “Older Adult”[Title/Abstract] OR “Older Person”[Title/Abstract] OR “Elderly”[Title/Abstract] OR “Elderly People”[Title/Abstract] OR “Veteran”[Title/Abstract] OR “Retired”[Title/Abstract] OR “Senior”[Title/Abstract] OR “Older People”[Title/Abstract] OR “Elder Person”[Title/Abstract] OR “Gerontology”[Title/Abstract])) AND (“Technology”[Mesh] OR “Technology”[Title/Abstract] OR “Computers”[Mesh] OR “Computers”[Title/Abstract] OR “wearable electronic devices”[Mesh] OR “wearable electronic devices” [Tittle/Abstract] OR “digital games”[Tittle/Abstract] OR “technologies”[Tittle/Abstract]) AND (English[lang] OR Portuguese[lang] OR Spanish[lang]) AND (Clinical Trial[ptyp] OR Review[ptyp] OR systematic[sb] OR Journal Article[ptyp] OR Congress[ptyp]) | 7 | 08/01/2020 |
| Cochrane | ( ( “Occupational Therapy” OR ergotherapy) AND ( “Accidental Falls” OR “Falls” ) AND ( “Aged” OR “Geriatrics” OR “Older Adult” OR “Older Person” OR “Elderly” OR “Elderly People” OR “Veteran” OR “Retired” OR “Senior” OR “Older People” OR “Elder Person” OR “Gerontology” ) AND ( “Technolog*” OR “Computers” OR “Wearables” OR “Digital games” ) ) en Título Resumen Palabra clave - (Se han buscado variaciones de la palabra) | 2 | 08/01/2020 |
| Scopus | ( TITLE-ABS-KEY ( “Occupational Therapy” OR ergotherapy) AND TITLE-ABS-KEY ( “Accidental Falls” OR “Falls” ) AND TITLE-ABS-KEY ( “Aged” OR “Geriatrics” OR “Older Adult” OR “Older Person” OR “Elderly” OR “Elderly People” OR “Veteran” OR “Retired” OR “Senior” OR “Older People” OR “Elder Person” OR “Gerontology” ) AND TITLE-ABS-KEY ( “Technolog*” OR “Computers” OR “Wearables” OR “Digital games” ) ) AND ( LIMIT-TO ( DOCTYPE , “ar” ) OR LIMIT-TO ( DOCTYPE , “re” ) OR LIMIT-TO ( DOCTYPE , “cp” ) ) AND ( LIMIT-TO ( LANGUAGE , “English” ) ) | 17 | 08/01/2020 |
| Web of Science | TEMA: (“Occupational therapy” OR ergotherapy) AND TEMA: (“Accidental falls” OR Falls) AND TEMA: (Aged OR Geriatrics OR “Older adult” OR “Older person” OR Elderly OR “Elderly people” OR Veteran OR Retired OR Senior OR “Older people” OR “Elder person” OR “Gerontology”) AND TEMA: (Technolog* OR Computers OR Wearables OR “Digital games”) | 25 | 08/01/2020 |
| BVS | "terapia ocupacional" AND caídas AND mayores AND tecnología | 6 | 08/01/2020 |
| OTSeeker | "occupational therapy" AND "falls" AND "technology" | 1 | 08/01/2020 |

**Table S3.** Removed from eligibility criteria.

|  | **Tittle** | **Abstract** | **Full Text** |
| --- | --- | --- | --- |
| 1. The Emergency Department Stopping Elderly Accidents, Deaths and Injuries Program - Full Text View - ClinicalTrials.gov. (n.d.). Retrieved January 8, 2020, from https://clinicaltrials.gov/ct2/show/NCT02167737. |  | Occupational therapy, falls and technology are not included |  |
| 1. Proceedings of the 3rd IPLeiria’s International Health Congress: Leiria, Portugal. 6-7 May 2016. (2016). BMC Health Services Research, 16 Suppl 3, 200. https://doi.org/10.1186/s12913-016-1423-5. | This paper are not available |  |  |
| 1. Effectiveness of one home visit by an occupational therapist in the prevention of falls: A quasi-randomized controlled trial in elderly women who sustained a hip fracture Fonte: Calcified Tissue International [0171-967X] Di Monaco, M ano: 2008 vol:82 px:S222 -S222. (2008). Retrieved from https://link.springer.com/journal/volumesAndIssues/223. |  | Technology is not included |  |
| 1. Bathroom safety: Environmental modifications to enhance bathing and aging in place in the elderly. (n.d.). Retrieved January 8, 2020, from https://www.researchgate.net/publication/289860337_Bathroom_safety_Environmental_modifications_to_enhance_bathing_and_aging_in_place_in_the_elderly. |  | Technology is not included |  |
| 1. Arthanat, S., Wilcox, J., & Macuch, M. (2019). Profiles and Predictors of Smart Home Technology Adoption by Older Adults. OTJR Occupation, Participation and Health, 39(4), 247–256. https://doi.org/10.1177/1539449218813906. |  |  | Accepted |
| 1. Ben Haj Khaled, A., Khalfallah, A., & Bouhlel, M. S. (2020). Fall Prevention Exergame Using Occupational Therapy Based on Kinect. Smart Innovation, Systems and Technologies, 146, 479–493. https://doi.org/10.1007/978-3-030-21005-2_46. |  |  | Accepted |
| 1. Bernardo, L. D. (2018). Older adults with Alzheimer’s disease: A systematic review about the Occupational Therapy intervention in changes of performance skills. Brazilian Journal of Occupational Therapy, 26(4), 926–942. https://doi.org/10.4322/2526-8910.ctoAR1066. |  |  | Falls are not included |
| 1. Bleijlevens, M. H. C., Hendriks, M. R. C., Van Haastregt, J. C. M., Crebolder, H. F. J. M., & Van Eijk, J. T. M. (2010). Lessons learned from a multidisciplinary fall-prevention programme: The occupational-therapy element. Scandinavian Journal of Occupational Therapy, 17(4), 319–325. https://doi.org/10.3109/11038120903419038. |  | Technology is not included |  |
| 1. Bleijlevens, M. H., Hendriks, M. R., van Haastregt, J. C., van Rossum, E., Kempen, G. I., Diederiks, J. P., … van Eijk, J. T. (2008). Process factors explaining the ineffectiveness of a multidisciplinary fall prevention programme: A process evaluation. BMC Public Health, 8(1), 332. https://doi.org/10.1186/1471-2458-8-332. |  | Technology is not included |  |
| 1. Briggs, R., & O’Neill, D. (2014, March 1). Vascular gait dyspraxia. Clinical Medicine, Journal of the Royal College of Physicians of London, Vol. 14, pp. 200–202. https://doi.org/10.7861/clinmedicine.14-2-200. | Falls, older adults, technology and occupational therapy are not included |  |  |
| 1. Chang, Y. W., Chang, Y. H., Pan, Y. L., Kao, T. W., & Kao, S. (2017). Validation and reliability of Falls Risk for Hospitalized Older People (FRHOP). Medicine (United States), 96(31). https://doi.org/10.1097/MD.0000000000007693. |  | Technology is not included |  |
| 1. Charness, N. (2014). Utilizing Technology to Improve Older Adult Health. Occupational Therapy In Health Care, 28(1), 21–30. https://doi.org/10.3109/07380577.2013.865859. |  |  | Accepted |
| 1. Chase, C. A., Mann, K., Wasek, S., & Arbesman, M. (2012). Systematic Review of the Effect of Home Modification and Fall Prevention Programs on Falls and the Performance of Community-Dwelling Older Adults. American Journal of Occupational Therapy, 66(3), 284–291. https://doi.org/10.5014/ajot.2012.005017. |  |  | Accepted |
| 1. Connell, B. R. (1996). Role of the environment in falls prevention. Clinics in Geriatric Medicine, Vol. 12, pp. 859–880. https://doi.org/10.1016/s0749-0690(18)30205-2. |  | Technology is not included |  |
| 1. Faes, M. C., Reelick, M. F., Esselink, R. A., & Rikkert, M. G. O. (2010, November). Developing and evaluating complex healthcare interventions in geriatrics: The use of the medical research council framework exemplified on a complex fall prevention intervention. Journal of the American Geriatrics Society, Vol. 58, pp. 2212–2221. https://doi.org/10.1111/j.1532-5415.2010.03108.x. |  | Occupational therapy is not included |  |
| 1. Ganesh, S., Hayter, A., Kim, J., Sanford, J., Sprigle, S., & Hoenig, H. (2007). Wheelchair Use by Veterans Newly Prescribed a Manual Wheelchair. Archives of Physical Medicine and Rehabilitation, 88(4), 434–439. https://doi.org/10.1016/j.apmr.2006.12.045. | Falls are not included |  |  |
| 1. Gately, M. E., Trudeau, S. A., & Moo, L. R. (2019). Feasibility of Telehealth-Delivered Home Safety Evaluations for Caregivers of Clients With Dementia. OTJR Occupation, Participation and Health. https://doi.org/10.1177/1539449219859935. |  | Occupational therapy is not included |  |
| 1. Gaugler, J. E., & Kane, R. L. (2015). Family Caregiving in the New Normal. In Family Caregiving in the New Normal. https://doi.org/10.1093/geront/gnv333.06. | Falls, older adults, occupational therapy and technology are not included |  |  |
| 1. Glännfjord, F., Hemmingsson, H., & Larsson Ranada, Å. (2017). Elderly people’s perceptions of using Wii sports bowling–A qualitative study. Scandinavian Journal of Occupational Therapy, 24(5), 329–338. https://doi.org/10.1080/11038128.2016.1267259. |  |  | Accepted |
| 1. Hamm, J., Money, A. G., & Atwal, A. (2019). Enabling older adults to carry out paperless falls-risk self-assessments using guidetomeasure-3D: A mixed methods study. 92, 103135. https://doi.org/10.1016/j.jbi.2019.103135. |  |  | Accepted |
| 1. Hamm, J., Money, A. G., Atwal, A., & Ghinea, G. (2019). Mobile three-dimensional visualisation technologies for clinician-led fall prevention assessments. Health Informatics Journal, 25(3), 788–810. https://doi.org/10.1177/1460458217723170. |  |  | Accepted |
| 1. Horowitz, B. P., Nochajski, S. M., & Schweitzer, J. A. (2013). Occupational therapy community practice and home assessments: use of the home safety self-assessment tool (HSSAT) to support aging in place. Occupational Therapy in Health Care, 27(3), 216–227. https://doi.org/10.3109/07380577.2013.807450. |  |  | Accepted |
| 1. Intiso, D., Di Rienzo, F., Russo, M., Pazienza, L., Tolfa, M., Iarossi, A., & Maruzzi, G. (2012). Rehabilitation strategy in the elderly. Journal of Nephrology, 25(SUPPL.19). https://doi.org/10.5301/jn.5000138. |  | Occupational therapy is not included |  |
| 1. King, E. C., & Novak, A. C. (2017). Effect of bathroom AIDS and age on balance control during bathing transfers. American Journal of Occupational Therapy, 71(6). https://doi.org/10.5014/ajot.2017.027136. |  | Technology is not included |  |
| 1. Krishnan, S., Pappadis, M. R., Weller, S. C., Fisher, S. R., Hay, C. C., & Reistetter, T. A. (2018). Patient-centered mobility outcome preferences according to individuals with stroke and caregivers: a qualitative analysis. Disability and Rehabilitation, 40(12), 1401–1409. https://doi.org/10.1080/09638288.2017.1297855. |  | Occupational therapy, falls, older adults and technology is not included |  |
| 1. Layton, N., Clarke, A., & Pennock, J. (2014, December 1). “Doing with not doing for”: a paradigm shift in home care services and what it means for occupational therapy. Australian Occupational Therapy Journal, Vol. 61, pp. 11–13. https://doi.org/10.1111/1440-1630.12184. |  | Technology is not included |  |
| 1. Lemmens, R., Gielen, C., & Spooren, A. A tool to assess. , 242 § (2017). |  |  | Accepted |
| 1. Lo Bianco, M., Pedell, S., & Renda, G. (2016). Augmented reality and home modifications: A tool to empower older adults in fall prevention. Proceedings of the 28th Australian Computer-Human Interaction Conference, OzCHI 2016, 499–507. https://doi.org/10.1145/3010915.3010929. |  |  | Accepted |
| 1. Mackenzie, L., & Clifford, A. (2020). Perceptions of older people in Ireland and Australia about the use of technology to address falls prevention. Ageing and Society, 40(2), 369–388. https://doi.org/10.1017/S0144686X18000983. |  | Occupational therapy is not included |  |
| 1. Mao, H. F., Chang, L. H., Tsai, A. Y. J., Huang, W. N., & Wang, J. (2016). Developing a referral protocol for community-based occupational therapy services in Taiwan: A logistic regression analysis. PLoS ONE, 11(2). https://doi.org/10.1371/journal.pone.0148414. |  | Technology is not included |  |
| 1. Mengshoel, A. M., & Skarbø, Å. (2017). Rehabilitation needs approached by health professionals at a rheumatism hospital. Musculoskeletal Care, 15(3), 210–217. https://doi.org/10.1002/msc.1162. | Older adults, falls, occupational therapy and technology are not included |  |  |
| 1. Money, A. G., Atwal, A., Boyce, E., Gaber, S., Windeatt, S., & Alexandrou, K. (2019). Falls Sensei: A serious 3D exploration game to enable the detection of extrinsic home fall hazards for older adults. BMC Medical Informatics and Decision Making, 19(1). https://doi.org/10.1186/s12911-019-0808-x. |  |  | Accepted |
| 1. Pighills, A., Drummond, A., Crossland, S., & Torgerson, D. J. (2019). What type of environmental assessment and modification prevents falls in community dwelling older people? BMJ (Online), 364. https://doi.org/10.1136/bmj.l880. |  | Technology is not included |  |
| 1. Plow, M., & Finlayson, M. (2014). A qualitative study exploring the usability of nintendo wii fit among persons with multiple sclerosis. Occupational Therapy International, 21(1), 21–32. https://doi.org/10.1002/oti.1345. | Older adults are not included |  |  |
| 1. Roach, J., Singh, J., & Pusalkar, P. (2012). Elderly patients with conservatively managed subdural haemorrhage should have a follow-up plan. QJM, 105(12), 1201–1203. https://doi.org/10.1093/qjmed/hcr140. | Occupational therapy, technology and falls are not included |  |  |
| 1. Sanders, M. J., O’Sullivan, B., DeBurra, K., & Fedner, A. (2013). Computer Training for Seniors: An Academic-Community Partnership. Educational Gerontology, 39(3), 179–193. https://doi.org/10.1080/03601277.2012.700816. |  | Falls are not included |  |
| 1. Sheffield, C., Smith, C. A., & Becker, M. (2013). Evaluation of an agency-based occupational therapy intervention to facilitate aging in place. Gerontologist, 53(6), 907–918. https://doi.org/10.1093/geront/gns145. |  | Technology is not included |  |
| 1. Sipilä, S., Tirkkonen, A., Hänninen, T., Laukkanen, P., Alen, M., Fielding, R. A., … Törmäkangas, T. (2018). Promoting safe walking among older people: The effects of a physical and cognitive training intervention vs. physical training alone on mobility and falls among older community-dwelling men and women (the PASSWORD study): Design and methods of a randomized controlled trial. BMC Geriatrics, 18(1). https://doi.org/10.1186/s12877-018-0906-0. |  | Occupational therapy and technology are not included |  |
| 1. Somerville, E., Smallfield, S., Stark, S., Seibert, C., Arbesman, M., & Lieberman, D. (2016). Occupational Therapy Home Modification Assessment and Intervention. American Journal of Occupational Therapy, 70(5), 7005395010p1. https://doi.org/10.5014/ajot.2016.705002. |  | Technology is not included |  |
| 1. Steultjens, E. M. J., Dekker, J., Bouter, L. M., Jellema, S., Bakker, E. B., & van den Ende, C. H. M. (2004, September). Occupational therapy for community dwelling elderly people: A systematic review. Age and Ageing, Vol. 33, pp. 453–460. https://doi.org/10.1093/ageing/afh174. |  | Technology and falls are not included |  |
| 1. Stewart, L. S. P., & McKinstry, B. (2012). Fear of Falling and the Use of Telecare by Older People. British Journal of Occupational Therapy, 75(7), 304–312. https://doi.org/10.4276/030802212X13418284515758. |  |  | Accepted |
| 1. Wahl, H. W., Fänge, A., Oswald, F., Gitlin, L. N., & Iwarsson, S. (2009). The home environment and disability-related outcomes in aging individuals: What is the empirical evidence? Gerontologist, 49(3), 355–367. https://doi.org/10.1093/geront/gnp056. |  | Technology is not included |  |
