# Supplementary material for: Occupational Therapy and the Use of Technology on Older Adult Fall Prevention: A Scoping Review
Source: Int J Environ Res Public Health. 2021 Jan 15;18(2):702. doi: 10.3390/ijerph18020702 (PMC7830762; doi:10.3390/ijerph18020702)
Supplement: Supplementary file 1 [file ijerph-18-00702-s001.zip › ijerph-1017994-ES-SI-supplementary/Supplementary tables S2-3.pdf]

Supplementary materials

Table S2. Search strategies from each database.

Database	Search Term Used	Results	Date
CINAHL	((“Occupational Therapy” OR ergotherapy) AND (“Accidental Falls” OR “Falls”) AND (“Aged” OR “Geriatrics” OR “Older Adult” OR “Older Person” OR “Elderly” OR “Elderly People” OR “Veteran” OR “Retired” OR “Senior” OR “Older People” OR “Elder Person” OR “Gerontology”) AND (“Technolog*” OR “Computers” OR “Wearables” OR “Digital games”)) Language = English	15	08/01/2020
PubMed	(((“Occupational Therapy”[Mesh] OR “Occupational therapy”[Title/Abstract] OR ergotherapy [Title/Abstract])) AND (“Accidental Falls”[Mesh] OR “Accidental Falls”[Title/Abstract] OR “Falls”[Title/Abstract])) AND (“Aged”[Mesh] OR “Aged”[Title/Abstract] OR “Geriatrics”[Mesh] OR “Geriatrics”[Title/Abstract] OR “Older Adult”[Title/Abstract] OR “Older Person”[Title/Abstract] OR “Elderly”[Title/Abstract] OR “Elderly People”[Title/Abstract] OR “Veteran”[Title/Abstract] OR “Retired”[Title/Abstract] OR “Senior”[Title/Abstract] OR “Older People”[Title/Abstract] OR “Elder Person”[Title/Abstract] OR “Gerontology”[Title/Abstract])) AND (“Technology”[Mesh] OR “Technology”[Title/Abstract] OR “Computers”[Mesh] OR “Computers”[Title/Abstract] OR “wearable electronic devices”[Mesh] OR “wearable electronic devices” [Tittle/Abstract] OR “digital games”[Tittle/Abstract] OR “technologies”[Tittle/Abstract]) AND (English[lang] OR Portuguese[lang] OR Spanish[lang]) AND (Clinical Trial[ptyp] OR Review[ptyp] OR systematic[sb] OR Journal Article[ptyp] OR Congress[ptyp])	7	08/01/2020
Cochrane	((“Occupational Therapy” OR ergotherapy) AND (“Accidental Falls” OR “Falls”) AND (“Aged” OR “Geriatrics” OR “Older Adult” OR “Older Person” OR “Elderly” OR “Elderly People” OR “Veteran” OR “Retired” OR “Senior” OR “Older People” OR “Elder Person” OR “Gerontology”) AND (“Technolog*” OR “Computers” OR “Wearables” OR “Digital games”)) en Título Resumen Palabra clave - (Se han buscado variaciones de la palabra)	2	08/01/2020
Scopus	(TITLE-ABS-KEY (“Occupational Therapy” OR ergotherapy) AND TITLE-ABS-KEY (“Accidental Falls” OR “Falls”) AND TITLE-ABS-KEY (“Aged” OR “Geriatrics” OR “Older Adult” OR “Older Person” OR “Elderly” OR “Elderly People” OR “Veteran” OR “Retired” OR “Senior” OR “Older People” OR “Elder Person” OR “Gerontology”) AND TITLE-ABS-KEY (“Technolog*” OR “Computers” OR “Wearables” OR “Digital games”)) AND (LIMIT-TO (DOCTYPE , “ar”) OR LIMIT-TO (DOCTYPE , “re”) OR LIMIT-TO (DOCTYPE , “cp”)) AND (LIMIT-TO (LANGUAGE , “English”))	17	08/01/2020
Web of Science	TEMA: (“Occupational therapy” OR ergotherapy) AND TEMA: (“Accidental falls” OR Falls) AND TEMA: (Aged OR Geriatrics OR “Older adult” OR “Older person” OR Elderly OR “Elderly people” OR Veteran OR Retired OR Senior OR “Older people” OR “Elder person” OR “Gerontology”) AND TEMA: (Technolog* OR Computers OR Wearables OR “Digital games”)	25	08/01/2020
BVS	"terapia ocupacional" AND caídas AND mayores AND tecnología	6	08/01/2020
OTSeeker	"occupational therapy" AND "falls" AND "technology"	1	08/01/2020

Table S3. Removed from eligibility criteria.

	Tittle	Abstract	Full Text
1. The Emergency Department Stopping Elderly Accidents, Deaths and Injuries Program - Full Text View - ClinicalTrials.gov. (n.d.). Retrieved January 8, 2020, from https://clinicaltrials.gov/ct2/show/NCT02167737.		Occupational therapy, falls and technology are not included	
2. Proceedings of the 3rd IPLeiria's International Health Congress: Leiria, Portugal. 6-7 May 2016. (2016). BMC Health Services Research, 16 Suppl 3, 200. https://doi.org/10.1186/s12913-016-1423-5.	This paper are not available		
3. Effectiveness of one home visit by an occupational therapist in the prevention of falls: A quasi-randomized controlled trial in elderly women who sustained a hip fracture Fonte: Calcified Tissue International [0171-967X] Di Monaco, M ano: 2008 vol:82 px:S222 - S222. (2008). Retrieved from https://link.springer.com/journal/volumesAndIssues/223.		Technology is not included	
4. Bathroom safety: Environmental modifications to enhance bathing and aging in place in the elderly. (n.d.). Retrieved January 8, 2020, from https://www.researchgate.net/publication/289860337_Bathroom_safety_Environmental_modifications_to_enhance_bathing_and_aging_in_place_in_the_elderly.		Technology is not included	
5. Arthanat, S., Wilcox, J., & Macuch, M. (2019). Profiles and Predictors of Smart Home Technology Adoption by Older Adults. OTJR Occupation, Participation and Health, 39(4), 247–256. https://doi.org/10.1177/1539449218813906.			Accepted
6. Ben Haj Khaled, A., Khalfallah, A., & Bouhlel, M. S. (2020). Fall Prevention Exergame Using Occupational Therapy Based on Kinect. Smart Innovation, Systems and Technologies, 146, 479–493. https://doi.org/10.1007/978-3-030-21005-2_46.			Accepted
7. Bernardo, L. D. (2018). Older adults with Alzheimer's disease: A systematic review about the Occupational Therapy intervention in changes of performance skills. Brazilian Journal of Occupational Therapy, 26(4), 926–942. https://doi.org/10.4322/2526-8910.ctoAR1066.			Falls are not included
8. Bleijlevens, M. H. C., Hendriks, M. R. C., Van Haastregt, J. C. M., Crebolder, H. F. J. M., & Van Eijk, J. T. M. (2010). Lessons learned from a multidisciplinary fall-prevention programme: The occupational-therapy element. Scandinavian Journal of Occupational Therapy, 17(4), 319–325. https://doi.org/10.3109/11038120903419038.		Technology is not included	
9. Bleijlevens, M. H., Hendriks, M. R., van Haastregt, J. C., van Rossum, E., Kempen, G. I., Diederiks, J. P., … van Eijk, J. T. (2008). Process factors explaining the ineffectiveness of a multidisciplinary fall prevention programme: A process evaluation. BMC Public Health, 8(1), 332. https://doi.org/10.1186/1471-2458-8-332.		Technology is not included	
10. Briggs, R., & O'Neill, D. (2014, March 1). Vascular gait dyspraxia. Clinical Medicine, Journal of the Royal College of Physicians of London, Vol. 14, pp. 200–202. https://doi.org/10.7861/clinmedicine.14-2-200.	Falls, older adults,		

	technology and occupational therapy are not included		
11. Chang, Y. W., Chang, Y. H., Pan, Y. L., Kao, T. W., & Kao, S. (2017). Validation and reliability of Falls Risk for Hospitalized Older People (FRHOP). Medicine (United States), 96(31). https://doi.org/10.1097/MD.0000000000007693.		Technology is not included	
12. Charness, N. (2014). Utilizing Technology to Improve Older Adult Health. Occupational Therapy In Health Care, 28(1), 21–30. https://doi.org/10.3109/07380577.2013.865859.			Accepted
13. Chase, C. A., Mann, K., Wasek, S., & Arbesman, M. (2012). Systematic Review of the Effect of Home Modification and Fall Prevention Programs on Falls and the Performance of Community-Dwelling Older Adults. American Journal of Occupational Therapy, 66(3), 284–291. https://doi.org/10.5014/ajot.2012.005017.			Accepted
14. Connell, B. R. (1996). Role of the environment in falls prevention. Clinics in Geriatric Medicine, Vol. 12, pp. 859–880. https://doi.org/10.1016/s0749-0690(18)30205-2.		Technology is not included	
15. Faes, M. C., Reelick, M. F., Esselink, R. A., & Rikkert, M. G. O. (2010, November). Developing and evaluating complex healthcare interventions in geriatrics: The use of the medical research council framework exemplified on a complex fall prevention intervention. Journal of the American Geriatrics Society, Vol. 58, pp. 2212–2221. https://doi.org/10.1111/j.1532-5415.2010.03108.x.		Occupational therapy is not included	
16. Ganesh, S., Hayter, A., Kim, J., Sanford, J., Sprigle, S., & Hoenig, H. (2007). Wheelchair Use by Veterans Newly Prescribed a Manual Wheelchair. Archives of Physical Medicine and Rehabilitation, 88(4), 434–439. https://doi.org/10.1016/j.apmr.2006.12.045.	Falls are not included		
17. Gately, M. E., Trudeau, S. A., & Moo, L. R. (2019). Feasibility of Telehealth-Delivered Home Safety Evaluations for Caregivers of Clients With Dementia. OTJR Occupation, Participation and Health. https://doi.org/10.1177/1539449219859935.		Occupational therapy is not included	
18. Gaugler, J. E., & Kane, R. L. (2015). Family Caregiving in the New Normal. In Family Caregiving in the New Normal. https://doi.org/10.1093/geront/gnv333.06.	Falls, older adults, occupational therapy and technology are not included		
19. Glännfjord, F., Hemmingsson, H., & Larsson Ranada, Å. (2017). Elderly people's perceptions of using Wii sports bowling–A qualitative study. Scandinavian Journal of Occupational Therapy, 24(5), 329–338. https://doi.org/10.1080/11038128.2016.1267259.			Accepted
20. Hamm, J., Money, A. G., & Atwal, A. (2019). Enabling older adults to carry out paperless falls-risk self-assessments using guidetomeasure-3D: A mixed methods study. 92, 103135. https://doi.org/10.1016/j.jbi.2019.103135.			Accepted

21. Hamm, J., Money, A. G., Atwal, A., & Ghinea, G. (2019). Mobile three-dimensional visualisation technologies for clinician-led fall prevention assessments. Health Informatics Journal, 25(3), 788–810. https://doi.org/10.1177/1460458217723170.		Accepted
22. Horowitz, B. P., Nochajski, S. M., & Schweitzer, J. A. (2013). Occupational therapy community practice and home assessments: use of the home safety self-assessment tool (HSSAT) to support aging in place. Occupational Therapy in Health Care, 27(3), 216–227. https://doi.org/10.3109/07380577.2013.807450.		Accepted
23. Intiso, D., Di Rienzo, F., Russo, M., Pazienza, L., Tolfa, M., Iarossi, A., & Maruzzi, G. (2012). Rehabilitation strategy in the elderly. Journal of Nephrology, 25(SUPPL.19). https://doi.org/10.5301/jn.5000138.	Occupational therapy is not included	
24. King, E. C., & Novak, A. C. (2017). Effect of bathroom AIDS and age on balance control during bathing transfers. American Journal of Occupational Therapy, 71(6). https://doi.org/10.5014/ajot.2017.027136.	Technology is not included	
25. Krishnan, S., Pappadis, M. R., Weller, S. C., Fisher, S. R., Hay, C. C., & Reistetter, T. A. (2018). Patient-centered mobility outcome preferences according to individuals with stroke and caregivers: a qualitative analysis. Disability and Rehabilitation, 40(12), 1401–1409. https://doi.org/10.1080/09638288.2017.1297855.	Occupational therapy, falls, older adults and technology is not included	
26. Layton, N., Clarke, A., & Pennock, J. (2014, December 1). "Doing with not doing for": a paradigm shift in home care services and what it means for occupational therapy. Australian Occupational Therapy Journal, Vol. 61, pp. 11–13. https://doi.org/10.1111/1440-1630.12184.	Technology is not included	
27. Lemmens, R., Gielen, C., & Spooren, A. A tool to assess. , 242 § (2017).		Accepted
28. Lo Bianco, M., Pedell, S., & Renda, G. (2016). Augmented reality and home modifications: A tool to empower older adults in fall prevention. Proceedings of the 28th Australian Computer-Human Interaction Conference, OzCHI 2016, 499–507. https://doi.org/10.1145/3010915.3010929.		Accepted
29. Mackenzie, L., & Clifford, A. (2020). Perceptions of older people in Ireland and Australia about the use of technology to address falls prevention. Ageing and Society, 40(2), 369–388. https://doi.org/10.1017/S0144686X18000983.	Occupational therapy is not included	
30. Mao, H. F., Chang, L. H., Tsai, A. Y. J., Huang, W. N., & Wang, J. (2016). Developing a referral protocol for community-based occupational therapy services in Taiwan: A logistic regression analysis. PLoS ONE, 11(2). https://doi.org/10.1371/journal.pone.0148414.	Technology is not included	
31. Mengshoel, A. M., & Skarbø, Å. (2017). Rehabilitation needs approached by health professionals at a rheumatism hospital. Musculoskeletal Care, 15(3), 210–217. https://doi.org/10.1002/msc.1162.	Older adults, falls, occupational therapy and technology	

	are not included		
32. Money, A. G., Atwal, A., Boyce, E., Gaber, S., Windeatt, S., & Alexandrou, K. (2019). Falls Sensei: A serious 3D exploration game to enable the detection of extrinsic home fall hazards for older adults. BMC Medical Informatics and Decision Making, 19(1). https://doi.org/10.1186/s12911-019-0808-x.			Accepted
33. Pighills, A., Drummond, A., Crossland, S., & Torgerson, D. J. (2019). What type of environmental assessment and modification prevents falls in community dwelling older people? BMJ (Online), 364. https://doi.org/10.1136/bmj.l880.		Technology is not included	
34. Plow, M., & Finlayson, M. (2014). A qualitative study exploring the usability of nintendo wii fit among persons with multiple sclerosis. Occupational Therapy International, 21(1), 21–32. https://doi.org/10.1002/oti.1345.	Older adults are not included		
35. Roach, J., Singh, J., & Pusalkar, P. (2012). Elderly patients with conservatively managed subdural haemorrhage should have a follow-up plan. QJM, 105(12), 1201–1203. https://doi.org/10.1093/qjmed/hcr140.	Occupational therapy, technology and falls are not included		
36. Sanders, M. J., O'Sullivan, B., DeBurra, K., & Fedner, A. (2013). Computer Training for Seniors: An Academic-Community Partnership. Educational Gerontology, 39(3), 179–193. https://doi.org/10.1080/03601277.2012.700816.		Falls are not included	
37. Sheffield, C., Smith, C. A., & Becker, M. (2013). Evaluation of an agency-based occupational therapy intervention to facilitate aging in place. Gerontologist, 53(6), 907–918. https://doi.org/10.1093/geront/gns145.		Technology is not included	
38. Sipilä, S., Tirkkonen, A., Hänninen, T., Laukkanen, P., Alen, M., Fielding, R. A., … Törmäkangas, T. (2018). Promoting safe walking among older people: The effects of a physical and cognitive training intervention vs. physical training alone on mobility and falls among older community-dwelling men and women (the PASSWORD study): Design and methods of a randomized controlled trial. BMC Geriatrics, 18(1). https://doi.org/10.1186/s12877-018-0906-0.		Occupational therapy and technology are not included	
39. Somerville, E., Smallfield, S., Stark, S., Seibert, C., Arbesman, M., & Lieberman, D. (2016). Occupational Therapy Home Modification Assessment and Intervention. American Journal of Occupational Therapy, 70(5), 7005395010p1. https://doi.org/10.5014/ajot.2016.705002.		Technology is not included	
40. Steultjens, E. M. J., Dekker, J., Bouter, L. M., Jellema, S., Bakker, E. B., & van den Ende, C. H. M. (2004, September). Occupational therapy for community dwelling elderly people: A systematic review. Age and Ageing, Vol. 33, pp. 453–460. https://doi.org/10.1093/ageing/afh174.		Technology and falls are not included	
41. Stewart, L. S. P., & McKinstry, B. (2012). Fear of Falling and the Use of Telecare by Older People. British Journal of Occupational Therapy, 75(7), 304–312. https://doi.org/10.4276/030802212X13418284515758.			Accepted
42. Wahl, H. W., Fänge, A., Oswald, F., Gitlin, L. N., & Iwarsson, S. (2009). The home environment and disability-related outcomes in aging individuals: What is the empirical evidence? Gerontologist, 49(3), 355–367. https://doi.org/10.1093/geront/gnp056.		Technology is not included	
